# Cannabinoids for treatment of Alzheimer’s disease: moving toward the clinic

**DOI:** 10.3389/fphar.2014.00037

**Published:** 2014-03-05

**Authors:** Ester Aso, Isidre Ferrer

**Affiliations:** ^1^Institut de Neuropatologia, Servei d’Anatomia Patològica, Institut d’Investigació Biomèdica de Bellvitge-Hospital Universitari de Bellvitge, Universitat de Barcelona, L’Hospitalet de LlobregatSpain; ^2^Centro de Investigación Biomédica en Red de Enfermedades Neurodegenerativas, Instituto Carlos IIISpain

**Keywords:** cannabinoids, Alzheimer, CB_1_ receptor, CB_2_ receptor, β-amyloid, tau, oxidative stress, neuroinflammation

## Abstract

The limited effectiveness of current therapies against Alzheimer’s disease (AD) highlights the need for intensifying research efforts devoted to developing new agents for preventing or retarding the disease process. During the last few years, targeting the endogenous cannabinoid system has emerged as a potential therapeutic approach to treat Alzheimer. The endocannabinoid system is composed by a number of cannabinoid receptors, including the well-characterized CB_1_ and CB_2_ receptors, with their endogenous ligands and the enzymes related to the synthesis and degradation of these endocannabinoid compounds. Several findings indicate that the activation of both CB_1_ and CB_2_ receptors by natural or synthetic agonists, at non-psychoactive doses, have beneficial effects in Alzheimer experimental models by reducing the harmful β-amyloid peptide action and tau phosphorylation, as well as by promoting the brain’s intrinsic repair mechanisms. Moreover, endocannabinoid signaling has been demonstrated to modulate numerous concomitant pathological processes, including neuroinflammation, excitotoxicity, mitochondrial dysfunction, and oxidative stress. The present paper summarizes the main experimental studies demonstrating the polyvalent properties of cannabinoid compounds for the treatment of AD, which together encourage progress toward a clinical trial.

## INTRODUCTION

Alzheimer is an age-dependent neurodegenerative process distinct from normal aging and characterized morphologically by the presence of senile plaques, mainly composed of different species of fibrillar β-amyloid (Aβ) produced by the cleavage of the Aβ precursor protein (APP) due to β- and γ-secretases, and by the presence of neurofibrillary tangles, mostly composed of various isoforms of hyper-phosphorylated and nitrated tau protein (Duyckaerts and Dickson, 2011; Ferrer, 2012). One tendency of opinion proposes that Aβ triggers plaque formation, tau hyper-phosphorylation, and disease progression (Hardy, 2009). This may happens in a percentage of familial Alzheimer’s disease (AD) cases linked to mutations in genes encoding APP, and presenilin 1 and presenilin 2 which are enzymes involved in the cleavage of APP, or in Down syndrome (Bertram and Tanzi, 2011). However, tau hyper-phosphorylation precedes Aβ deposition in many cerebral regions in sporadic cases of AD (Braak and Braak, 1999).

Recent studies have shown that Aβ acts as a seed of new Aβ production and deposition under appropriate conditions (Frost and Diamond, 2010) and that abnormal tau promotes the production and deposition of hyper-phosphorylated tau under determinate experimental conditions (Clavaguera et al., 2009). Therefore, Aβ and hyper-phosphorylated tau promote the progression of the pathological process in an exponential way once these abnormal proteins are accumulated in the brain (Goedert et al., 2010; Ferrer, 2012).

In addition to these pathological hallmarks, multiple alterations converge in the pathogenesis of AD, including genetic and environmental factors. Vascular factors and concomitant pathologies worsen disease symptoms (Kovacs et al., 2008). Mitochondrial functional defects, increased production of reactive oxygen and nitrogen species (ROS and RNS), and damage to enzymes involved in energy metabolism are causative of nerve cell exhaustion (Pamplona et al., 2005; Ferrer, 2009; Sultana and Butterfield, 2010).

Altered lipid composition of membranes particularly lipid rafts (Martín et al., 2010), inflammatory responses (Akiyama et al., 2000), and altered production of trophic factors, neurotransmitter and neuromodulators, together with impaired function of degradation pathways such as those related to cytoplasmic proteolysis, autophagy, and ubiquitin–proteasome system play crucial roles as well (Keller et al., 2000; Ferrer, 2012; Selkoe, 2012).

Neurofibrillary tangles first appear in middle age in selected nuclei of the brain stem, later in the entorhinal cortex, and then extend to other parts of the brain (Braak and Braak, 1999; Simic et al., 2009). Senile plaques appear first in the orbitofrontal and temporal cortex and then extend to the whole cortex, diencephalic nuclei, and eventually to the cerebellum at terminal stages (Braak and Braak, 1999). Synaptic loss, reduced dendritic arbors, progressive isolation of remaining neurons and nerve cell loss occurs with disease progression, and affects multiple brain regions not only the cerebral cortex but also the amygdala, nuclei of the forebrain including Meynert nucleus, striatum, thalamus, and selected nuclei of the brain stem thus involving multiple neurotransmitter systems (Duyckaerts and Dickson, 2011).

Importantly, the progression from early stages of the neurodegenerative process to symptomatic stages may take decades, whereas once the cognitive impairment and dementia appear the disease progression is much more rapid. Therefore, Alzheimer is a relatively well-tolerated degenerative process during a long period of time, but it may have devastating effects once thresholds are crossed (Ferrer, 2012). These facts highlight the need to search for treatments that act on selective targets during the silent period of the disease, aimed at curbing or retarding disease progression toward dementia (Ferrer, 2012; Selkoe, 2012).

During the last few years, targeting the endogenous cannabinoid system (ECS) has emerged as a potential therapeutic approach to treat Alzheimer in such first stages. Endocannabinoid signaling has been demonstrated to modulate the main pathological processes occurring during the silent period of the neurodegenerative process, including protein misfolding, neuroinflammation, excitotoxicity, mitochondrial dysfunction, and oxidative stress. The present paper summarizes the experimental studies demonstrating the multi-faceted properties of cannabinoid compounds for the treatment of AD.

## THE ENDOGENOUS CANNABINOID SYSTEM

The last two decades of research have brought a tremendous improvement in knowledge of the endocannabinoid system components and functions under physiological and pathological conditions. This neuromodulatory system consists of cannabinoid receptors, endogenous ligands, and several enzymes responsible for their synthesis and degradation (Piomelli, 2003). To date, two subtypes of cannabinoid G_i/o_-coupled receptors, CB_1_ and CB_2_, have been fully characterized and cloned. However, cannabinoid compounds may also bind to other receptors, such as GPR55, peroxisome proliferator-activated receptors PPARα and PPARγ, and transient receptor potential vannilloid-1 (TRPV1) channels (Maccarrone et al., 2010; Pertwee et al., 2010). CB_1_ receptors are the most abundant G protein-coupled receptors in the central neural system, expressed in both neurons and glial cells, where they regulate important brain functions including cognition and memory, emotion, motor control, feeding, and pain perception (Wilson and Nicoll, 2002; Howlett, 2005). CB_1_ receptors are mostly located at the terminals of neurons of the central and peripheral nervous system where they act as modulators of excitatory and inhibitory neurotransmission. Moreover, CB_1_ receptors are also found in peripheral tissues, playing an important role in energy balance and metabolism (Silvestri and Di Marzo, 2013). CB_2_ receptors are localized in cells of the immune system and modulate the immune cell migration and the release of cytokines; within the nervous system CB_2_ receptors are mainly located in microglia (Cabral and Griffin-Thomas, 2009). Relatively low CB_2_ receptor expression has also recently been identified in some neurons (Van Sickle et al., 2005; Brusco et al., 2008; Onaivi et al., 2008). Further evidence of CB_2_ receptor expression in neurons comes from the observation that axonal damage in one cerebellar hemisphere induced the expression of CB_2_ receptors in contralateral precerebellar neurons; CB_2_ receptor agonist facilitated neuronal survival, whereas the selective PI3K inhibitor blocked CB_2_R effects on axotomized neurons (Viscomi et al., 2009). Most of the knowledge acquired about cannabinoid receptor pharmacology was made possible by the study of the mechanisms of action of numerous natural, but also synthetic, cannabinoid compounds. Among the natural cannabinoids, the most well-known are Δ^9^-tetrahydrocannabinol (Δ^9^-THC), the main psychoactive compound of the *Cannabis sativa* plant, and cannabidiol (CBD), which is devoid of any psychoactivity, both differing in cannabinoid receptor affinity and activity (Pertwee, 2008).

The characterization of CB_1_ and CB_2_ receptors permitted the discovered of endocannabinoids or cannabinoids produced and released by nerve cells. Endocannabinoids are lipid compounds of the eicosanoid family derived from the degradation of membrane phospholipids (Piomelli, 2003). The most representative are arachidonoylethanolamine (AEA), also named anandamide, and 2-arachidonoylglycerol (2-AG), although several others have also been identified, such as 2-arachidonylglyceryl ether (2-AGE), virodhamine, and *N*-arachidonyldopamine (Piomelli, 2003). Endocannabinoids act as neurotransmitters since they are synthesized and released by neurons, are able to bind and activate membrane receptors, and are inactivated by reuptake and enzymatic degradation within the cell. However, endocannabinoids have two fundamental characteristics that differentiate them from other neurotransmitters: they act as retrograde messengers and they do not accumulate in the interior of synaptic vesicles (Wilson and Nicoll, 2002). For several years, endocannabinoid compounds have been supposed to be exclusively synthesized on demand to act on cells located near their site of synthesis, and then to be rapidly inactivated by the action of specific degradation enzymes. However, recent studies have shown intracellular stores of anandamide in places other than synaptic vesicles as in adiposomes where it is sequestered and concentrated to higher levels than in the extracellular space (Hillard and Jarrahian, 2000; Maccarrone et al., 2010). Moreover, an active intracellular binding of anandamide to TRPV1 and PPARs suggest an additional role of anandamide as a second messenger in intracellular signaling (Maccarrone et al., 2010). This new scenario contemplates the possibility that anandamide may act as an autocrine/paracrine ligand of CB receptors but also as an intracellular ligand to TRPV1 and PPARs; moreover, the presence of extracellular anandamide transporters would point anandamide as an endocrine messenger (Maccarrone et al., 2010).

Interestingly, neuronal damage can increase the production of endocannabinoids, which may provide a defense mechanism against toxicity (Stella et al., 1997; Marsicano et al., 2003). In the case of AEA, the synthesis is performed from the phosphatidylethanolamine existing in the cell membrane by the successive action of 2 enzymes, the *N*-acetyltransferase and phospholipase D (PLD). AEA action is determined by two processes that limit their availability, (i) transport from the synaptic cleft into the cell by passive diffusion or by a selective transporter, and (ii) the hydrolysis caused by two enzyme systems, mainly the fatty acid amide hydrolase (FAAH) but also monoacylglycerol lipase (MAGL; Di Marzo et al., 1994). 2-AG is the most abundant endogenous cannabinoid in the brain and its concentration is about 200 times that of AEA (Stella et al., 1997). The formation of 2-AG is mediated by phospholipase C and diacylglycerol lipase (DAGL), and also produced on demand. In contrast to AEA, MAGL seems to be more involved in 2-AG degradation than FAAH (Dinh et al., 2002).

Interest in the role that ECS may play in neurodegenerative processes is based on findings revealing that the augmentation of cannabinoid tone contributes to brain homeostasis and neuron survival, suggesting that may offer protection against the deleterious consequences of pathogenic molecules.

## THE ENDOGENOUS CANNABINOID SYSTEM IN AD BRAINS

The analysis of human *post-mortem* samples revealed some alterations in ECS composition and signaling in AD brains, although the bestowal of such modifications in the pathophysiology of the disease remains to be elucidated. The modifications described for CB_1_ receptors in AD are ambiguous. Whereas some authors have reported a significant reduction in the CB_1_ levels in cortical areas and in neurons distant from senile plaques (Ramírez et al., 2005; Solas et al., 2013), others have described no changes in the expression, distribution, or availability of CB_1_ receptors in cortex and hippocampus in AD (Benito et al., 2003; Lee et al., 2010; Mulder et al., 2011; Ahmad et al., 2013) or have failed to dissociate CB_1_ receptor expression changes from normal aging (Westlake et al., 1994). No correlation between CB_1_ levels and any AD molecular marker or cognitive status has been found (Solas et al., 2013). In contrast, there is no controversy regarding the significant increase of CB_2_ levels in AD brains, mainly corresponding to receptors expressed on microglia surrounding senile plaques (Ramírez et al., 2005; Solas et al., 2013). Interestingly, expression levels of CB_2_ receptors correlates with Aβ_42_ levels and plaque deposition, although not with cognitive status (Solas et al., 2013), suggesting that such pathogenic events induce CB_2_ receptor expression. Additionally, both CB_1_ and CB_2_ cannabinoid receptors in the AD brain are nitrosylated, and this could contribute to the impaired coupling of these receptors to downstream effector signaling molecules (Ramírez et al., 2005).

A few studies addressed other components of ECS in AD human samples. The first study analyzing endocannabinoid levels reported no differences between AD patients and healthy controls in the plasmatic concentrations of AEA and 2-AG (Koppel et al., 2009). However, a recent lipidomic study in *post-mortem* brain samples revealed lower AEA levels in midfrontal and temporal cortices in AD compared to control subjects, which inversely correlated with the neurotoxic brain Aβ_42_ peptide levels and cognitive deficiencies recorded in these patients, suggesting a contribution for Aβ_42_-dependent AEA impairment to cognitive dysfunction (Jung et al., 2012). Moreover, some alterations have been found in the contents and/or activity of the enzymes related to endocannabinoid synthesis and degradation in AD brains. Thus, the endocannabinoid metabolizing enzyme FAAH is up-regulated in AD both neuritic plaque-associated glia (Benito et al., 2003) and in peripheral blood mononuclear cells (D’Addario et al., 2012), and this could participate in the increase of AEA degradation in the vicinity of the senile plaque. Such FAAH overexpression may have at least two harmful consequences in disease progression, (i) neuronal AEA availability limitation and (ii) increase of pro-inflammatory molecules induced by AEA metabolites such as arachidonic acid (Calder, 2005). An elegant study revealed altered 2-AG signaling during late stages of AD due to the combination of impaired MAGL recruitment and increased DAGL levels, which subsidize synapse silencing in AD (Mulder et al., 2011). The same study failed to detect changes in PLD, FAAH, or TRPV1 protein levels in total hippocampal homogenates.

## CLINICAL AND PRECLINICAL EVIDENCE OF THERAPEUTIC PROPERTIES OF CANNABINOIDS IN AD

Most of the evidence accumulated sustaining the potential therapeutic utility of cannabinoids in AD has been obtained by using cellular and animal models that mimic a variety of AD-related changes, and they will be discussed later on in this review. However, it is of note that the scarce clinical data available also support the beneficial effects of cannabinoid compounds for treating some behavioral symptoms related to AD. Only a few clinical trials and one case report are available on the topic so far. In all the cases an analog of Δ^9^-THC (nabilone or dronabinol) was tested. Interestingly, one clinical trial including 15 AD patients resulted in a decreased severity of altered behavior and an increase in the body weight in AD patients, who were previously refusing food, after 6 weeks of dronabinol treatment. Side effects associated with cannabinoid administration were limited to euphoria, somnolence, and tiredness, but these did not warrant discontinuation of therapy (Volicer et al., 1997). Similarly, two pilot studies including eight patients with dementia concluded with a reduction in night-time agitation and behavioral disturbances, without adverse effects during the trial period with dronabinol (Walther et al., 2006, 2011). In line with these observations, the use of the cannabinoid receptor agonist nabilone correlated with prompt and dramatic improvements in the severe agitation and aggressiveness exhibited by an advanced AD patient who was refractory to anti-psychotic and anxiolytic medications (Passmore, 2008). In spite of the low number of patients included in these trials and the fact that none of them evaluated cognitive or neurodegenerative markers, the positive behavioral results are promising and represent valuable, albeit limited, information, considering that no remarkable side effects were reported. However, the revision in 2009 of the Cochrane Dementia and Cognitive Improvement Group Specialized Register found no evidences of cannabinoid effectiveness in the improvement of behavior and other parameters of dementia, and suggested that more controlled trials are needed to assess the effectiveness of cannabinoids in the treatment of dementia (Krishnan et al., 2009).

### EFFECT OF CANNABINOIDS ON Aβ

Several *in vitro* and *in vivo* studies demonstrate that certain cannabinoid compounds confer neuroprotection against Aβ, as previously reported elsewhere (Ruiz-Valdepeñas et al., 2010). Some endocannabinoids such as AEA, 2-AG, and noladin ether, directly supplied to the cell culture or augmenting their availability through administration of endocannabinoid reuptake inhibitors, increased the viability of neurons after exposure to different toxic Aβ species (Milton, 2002; Chen et al., 2011; Harvey et al., 2012; Janefjord et al., 2013), and reduced Aβ-induced memory impairment in rats (van der Stelt et al., 2006). Similar positive results in the survival of neuronal cultures exposed to Aβ peptide were obtained with exogenous cannabinoids such as CBD (Iuvone et al., 2004; Janefjord et al., 2013), the selective CB_1_ receptor agonist arachidonyl-2-chloroethylamide (ACEA; Aso et al., 2012), the CB_2_ selective agonists JWH-015 and JWH-133, and the mixed CB_1_/CB_2_ receptor agonists Δ^9^-THC, HU-210, and WIN55,212-2 (Ramírez et al., 2005; Janefjord et al., 2013). The neuroprotective properties of exogenous cannabinoids have consistently been demonstrated to prevent memory deficits in Aβ-injected rats and mice for both synthetic CB_1_ (Haghani et al., 2012) and CB_2_ selective agonists (Wu et al., 2013), as well as mixed CB_1_/CB_2_ receptor agonists (Ramírez et al., 2005; Martín-Moreno et al., 2011; Fakhfouri et al., 2012) and natural CBD (Martín-Moreno et al., 2011). Moreover, chronic treatment with ACEA (Aso et al., 2012), JWH-133 (Martín-Moreno et al., 2012; Aso et al., 2013), or WIN55,212-2 (Martín-Moreno et al., 2012) resulted in cognitive improvement in two different transgenic mouse models of brain amyloidosis. The efficacy of the cannabinoid compounds in curbing the cognitive impairment was inversely proportional to the disease progression stage at the beginning of the treatment in the transgenic animals (Aso et al., 2012, 2013).

The mechanisms of action that underlie the cannabinoid neuroprotection against Aβ, which ultimately may lead to the memory improvement, are multiple and are assumed to act in parallel or interacting within them. Although most of these proposed protective mechanisms are related to the capacity of cannabinoids to indirectly mitigate the harmful effects of Aβ, as we will discuss in later sections of this review (i.e., inflammation, oxidative stress, excitotoxicity, aberrant cellular signaling), some authors also described direct effects of cannabinoids on Aβ processing. Thus, stimulation of CB_2_ receptors produced Aβ removal by human macrophages (Tolón et al., 2009; Wu et al., 2013) and favored Aβ transport through the choroid plexus (Martín-Moreno et al., 2012). This facilitation of Aβ clearance across the blood–brain barrier (BBB) was also demonstrated for the endocannabinoid 2-AG, a synthetic CB_1_/CB_2_ receptor agonist and MAGL inhibitors, but no FAAH, in *in vitro* an *in vivo* BBB models (Bachmeier et al., 2013). These findings could explain the reduction in Aβ levels and plaque burden observed in AD mouse models chronically treated with CB_2_ or CB_1_/CB_2_ receptor agonists (Martín-Moreno et al., 2012) and MAGL inhibitors (Chen et al., 2012; Piro et al., 2012). In contrast, no significant contribution of CB_1_ receptors in Aβ production, aggregation or clearance was reported after chronic treatment with ACEA (Aso et al., 2012) or HU-210 (Chen et al., 2010) in two different transgenic AD models. However, there is a study reporting a regulatory influence of CB_1_ receptor on APP processing since APP23 transgenic mice deficient for CB_1_ receptor exhibited reduced APP protein levels and Aβ plaque deposition, likely due to changes in intracellular APP transport, although the animals presented enhanced cognitive deficits (Stumm et al., 2013). Moreover, a recent publication revealed that Δ^9^-THC significantly increased the expression of neprilysin, an important endopeptidase for Aβ degradation, but not β-site APP cleaving enzyme 1 (BACE1), leading to a remarkable reduction of Aβ plaques in 5xFAD APP transgenic mice (Chen et al., 2013). This study failed, however, to clarify the specific role of CB_1_ or CB_2_ receptors in such Δ^9^-THC effect on Aβ clearance.

### CANNABINOIDS ON TAU HYPER-PHOSPHORYLATION

A role for cannabinoids in another AD pathological hallmark, tau hyper-phosphorylation, has also been described. First studies performed in cell cultures demonstrated that CBD, ACEA, and WIN55,212-2 inhibited tau protein hyper-phosphorylation in Aβ-stimulated PC12 neuronal cells (Esposito et al., 2006a,b). In the case of CBD, the effect was mediated through the reduction of the phosphorylated active form of glycogen synthase kinase 3β (GSK-3β), one of the known tau kinases (Ferrer et al., 2005), which in turn resulted in Wnt/β-catenin pathway rescue and consequent reduction of neuronal apoptosis (Esposito et al., 2006a). In contrast, the ACEA and WIN55,212-2 effect on tau hyper-phosphorylation was selectively mediated by CB_1_ receptor through the down-regulation of inducible nitric oxide synthase (iNOS) protein expression and nitric oxide (NO) production in Aβ-stimulated astroglioma cells co-cultured with the PC12 neuronal cells (Esposito et al., 2006b). In line with the described role for CB_1_ receptor on tau hyper-phosphorylation, chronic treatment with the CB_1_ selective agonist ACEA reduced the levels of tau phosphorylated at Thr181 site in the area surrounding Aβ plaques in treated APP/PS1 mice, probably through the ACEA-induced reduction in GSK-3β harmful activity (Aso et al., 2012). Moreover, a specific role for CB_2_ receptor in the modulation of tau phosphorylation was also suggested. Chronic JWH-133 administration reduced tau hyper-phosphorylation in the vicinity of Aβ plaques in APP/PS1 mice, which may be explained by decreased activity of GSK-3β, p38, and stress-activated protein kinase/c-Jun NH(2)-terminal kinase (SAPK/JNK) kinases in the treated animals (Aso et al., 2013).

Confirming these observations, a recent study reported a marked reduction in neurofibrillary tangles in parkin-null, human tau overexpressing (PK^-^^/^^-^/TauVLW) mice, a model of complex frontotemporal dementia, parkinsonism, and lower motor neuron disease, after prolonged exposure to Sativex^®^, an already approved medicine based on mixed Δ^9^-THC and CBD natural extracts (Casarejos et al., 2013). The authors suggested the cannabinoid potentiation of autophagy or improvement of redox state as likely mechanisms accounting for the reduction in tau deposition.

### ANTI-INFLAMMATORY PROPERTIES OF CANNABINOIDS

Neuroinflammation, initially manifested as microglial activation, is a prominent feature in AD which contributes to progressive cell damage and neuron loss (Akiyama et al., 2000; Hensley, 2010; Sardi et al., 2011). As CB_2_ receptors are essentially expressed in the immune system including microglial cells, where they are known to inhibit microglia-mediated neurotoxicity, the main interest in the role of cannabinoids as anti-inflammatory agents in several diseases concurring with inflammation has focused on compounds acting on CB_2_ receptors (Cabral and Griffin-Thomas, 2009). In the case of AD, several studies reported that activation of CB_2_ receptors reduced the neuroinflammatory response to Aβ insults in different models of the disease. After the inoculation of Aβ into the rat or mouse brain, selective or mixed CB_2_ receptor agonists reduced microglial response and pro-inflammatory molecule production in a plethora of studies (Ramírez et al., 2005; van der Stelt et al., 2006; Esposito et al., 2007; Fakhfouri et al., 2012; Wu et al., 2013). Similarly, selective CB_2_ receptor agonists decreased the number of activated microglial cells surrounding Aβ deposition and the levels of pro-inflammatory cytokines in at least two APP transgenic models (Martín-Moreno et al., 2012; Aso et al., 2013). Moreover, the Δ^9^-THC and CBD natural mixture present in Sativex^®^ also blunted the microglial reactivity in a genetic tauopathy model (Casarejos et al., 2013), although no evidence of direct implication of CB_2_ receptors or other receptors in such effects was provided. In fact, other mechanisms related to ECS components distinct from CB_2_ receptors could explain the anti-inflammatory effects of the Sativex^®^ preparation. As noted above, Δ^9^-THC is a partial agonist of CB_1_ receptors, which could also play a role in the AD inflammatory process according to a recent study demonstrating that chronic treatment with the selective agonist ACEA reduced the astrocytic expression of the pro-inflammatory cytokine interferon-γ in APP/PS1 transgenic mice (Aso et al., 2012). Additionally, CBD, which has no affinity for CB_1_ or CB_2_ receptors, also presents anti-inflammatory properties in AD models (Esposito et al., 2006a; Martín-Moreno et al., 2011). The precise site at which CBD could exert its neuroinflammatory and neuroprotective effects is still not fully elucidated, but some findings point to the selective involvement of PPARγ in such CBD properties (Esposito et al., 2011).

The enzymes related to AEA and 2-AG degradation also contribute to modulating the inflammatory process in AD models. FAAH is expressed in both neurons and astrocytes, where it may play a role in the response to inflammation. In fact, an astrocyte-specific increase in FAAH expression is markedly maintained in neuroinflammatory conditions including amyloidosis, which was assumed to contribute to the harmful processes induced by toxic insults because of the reduction in endocannabinoid tone (Benito et al., 2003). However, cortical mouse astrocytes genetically modified to lack FAAH exhibited a pro-inflammatory phenotype when exposed to Aβ, characterized by an increase in cytokine concentration and cell death probably due to the modification of signaling cascades involved in cell survival and inflammation, such as extracellular signal-regulated protein kinases 1 and 2 (ERK1/2), p38 mitogen-activated protein kinase (p38MAPK), and nuclear factor kappa-light-chain-enhancer of activated B cells (NF-κB), as well as to the increase in inflammatory mediators such as iNOS and cyclooxygenase (COX-2; Benito et al., 2012). The authors demonstrated that these processes involved PPAR-α, PPAR-γ, and TRPV1, but not CB_1_ or CB_2_ receptors. Yet, the pharmacological blockade of FAAH in cell cultures did not lead astrocytes to a pro-inflammatory phenotype, indicating that the observed effects in astrocytes lacking FAAH could be due to compensatory changes that result from the potentially prolonged enhancement of *N*-acylethanolamines. These data suggest that an excessively prolonged enhancement of the endocannabinoid tone may have harmful consequences. In contrast, the genetic inactivation of MAGL, an enzyme known to hydrolyze endocannabinoids and generate the primary arachidonic acid pool for neuroinflammatory prostaglandins (Nomura et al., 2011), attenuated neuroinflammation and lowered Aβ levels and plaques in APP/PS1 mice (Piro et al., 2012). These observations were confirmed by the pharmacological blockade of MAGL, which recapitulated the cytokine-lowering effects through reduced prostaglandin production, rather than enhanced endocannabinoid signaling.

### CANNABINOID ACTIONS ON MITOCHONDRIA ACTIVITY: OXIDATIVE STRESS AND ENERGY METABOLISM

Mitochondria are vital cellular components essential for ATP production and calcium homeostasis. The relevance of these organelles in neurons is even greater than in other cell types because neurons are highly demanding energy cells mainly dependent on aerobic oxidative phosphorylation, due to their limited capacity for glycolysis. Long axons require energy transport over long distances, and synaptic transmission depends on calcium signals. Mitochondria are abundant in presynaptic nerve terminals where they provide energy for sustained neurotransmitter release. Therefore, defects in mitochondrial activity can have severe consequences for the cell, including energetic failure associated with decreased ATP production and apoptosis resulting from the release of death factors and impaired calcium-buffering capacity. Moreover, alterations in the protein complexes of the respiratory chain located in the inner mitochondrial membrane lead to electron transport leakage that enables the production of ROS, which may overwhelm the capacity of the anti-oxidant systems existing in cells to counteract free radical damage, with the subsequent oxidative damage produced to proteins, DNA, RNA, and lipids. Numerous studies have linked mitochondrial dysfunction to neurodegenerative diseases, including AD (Ferrer, 2009; Ankarcrona et al., 2010; Burchell et al., 2010). Altered mitochondrial function appears early in time in AD, even preceding the characteristic Alzheimer pathology in mouse models, and ultimately leads to exhausted neurons as a result of the convergence of reduced energy production, increased energy demand, and excessive oxidative stress (Ferrer, 2009).

The anti-oxidant properties of cannabis derivatives, notably CBD, were demonstrated early in cell cultures exposed to toxic glutamate levels (Hampson et al., 1998, 2000). In line with these findings, CBD prevented ROS production and lipid peroxidation in PC12 neuronal cells exposed to Aβ, as well as reduced apoptosis from reduced caspase 3 levels, and in counteracting the Aβ-induced increase in intracellular calcium concentration (Iuvone et al., 2004). CBD also reduced, in similar conditions, the levels of nitrite (NO), a potent oxidant reactive molecule, as well as the expression of iNOS, one of the enzymes responsible for the synthesis of NO (Esposito et al., 2006b, 2011). Moreover, other cannabinoid compounds exhibited anti-oxidant properties in animal models of AD. Thus, the selective CB_2_ receptor agonist JWH-133 reduced hydroxynonenal adducts, derived from lipid peroxidation, and enhanced the levels of the superoxide dismutases SOD1 and SOD2 in the vicinity of plaques in APP/PS1 mice, indicating the role of CB_2_ receptors in reducing oxidative stress damage and to promote responses against such damage (Aso et al., 2013). A reduction of free radicals and mitochondrial activity was also suggested in a mouse model of tauopathy exposed to chronic treatment with the Sativex^®^ mixture of Δ^9^-THC and CBD (Casarejos et al., 2013).

An additional topic deserving attention is the putative role of cannabinoid receptors in the regulation of neuronal energy metabolism. The little information available to date supports the direct control of CB_1_ over neuronal respiration and energy production. One study, using anti-CB_1_ receptor antibodies, revealed CB_1_ receptor protein localization in approximately 30% of neuronal mitochondria, which when activated by exogenous or endogenous cannabinoids reduces the respiratory chain complex I activity and oxygen consumption, likely through cyclic adenosine monophosphate (cAMP) and protein kinase A (PKA) signaling (Bénard et al., 2012). These findings are in agreement with previous observations showing that AEA, Δ^9^-THC, and HU-210, all of them partial CB_1_ agonists, significantly decreased oxygen consumption and mitochondrial membrane potential (Athanasiou et al., 2007). However, these data must be interpreted with caution as commercial anti-CB_1_ receptor antibodies also recognize the mitochondrial protein stomatin-like protein 2 and that the formerly described effect of WIN 55,212-2 on mitochondrial complex III is in fact not detectable in isolated mitochondrial preparations (Morozov et al., 2013).

### CANNABINOIDS MODULATE NEUROTRANSMISSION

Nowadays the approved drugs for treating AD are based on acetylcholine esterase (AChE) inhibitors, which increase the ACh availability partially palliating this neurotransmitter deficiency in AD patients, or they are non-competitive antagonists of the *N*-methyl D-aspartate (NMDA) receptor, which reduce calcium influx and limit excitotoxicity. Interestingly, certain cannabinoid compounds act on the same targets than current medications, resulting in similar or enhanced beneficial effects. For instance, Δ^9^-THC competitively inhibits AChE, thus increasing ACh levels, as well as preventing AChE-induced Aβ aggregation by binding in the peripheral anionic site of AChE, the critical region involved in amyloidogenesis (Eubanks et al., 2006). The synthetic cannabinoid HU-211 acts as a stereoselective inhibitor of NMDA receptors, and thus protects cells from NMDA induced neurotoxicity (Feigenbaum et al., 1989; Eshhar et al., 1993; Nadler et al., 1993). In the case of HU-211, its neuroprotective activity is due to the direct binding to NMDA receptors, not to cannabinoid receptors, but the broadly accepted cannabinoid-mediated neuroprotection against excitotoxicity can be achieved through a number of other different mechanisms, including inhibition of presynaptic glutamate release (Marsicano et al., 2003), blockage of voltage-dependent calcium channels (Mackie and Hille, 1992; Twitchell et al., 1997) and inhibition of calcium release from ryanodine sensitive stores (Zhuang et al., 2005), which mostly imply the direct or indirect participation of CB_1_ receptors.

### OTHER EFFECTS OF CANNABINOIDS IN AD

Several other mechanisms seem to contribute to the therapeutic properties of cannabinoid compounds in AD, although they have not been fully characterized. Among them, we can note the capacity of endocannabinoids to prevent Aβ-mediated lysosomal destabilization in cultured neurons, reducing in this way the apoptotic signaling, which in turn sustains cell survival (Noonan et al., 2010). Compromised neurogenesis is an early event in AD that limits neuronal replacement once progressive neuronal loss takes place in the brain, contributing to cognitive deterioration (Lazarov and Marr, 2010). Interestingly, AEA and CBD have been described as promoting neurogenesis in response to Aβ insult (Esposito et al., 2011; Tanveer et al., 2012), suggesting an additional beneficial effect of cannabinoids in AD.

Another aspect as yet unexplored is the interaction of cannabinoids with neurotrophic factors in AD. Cannabinoids are capable of increasing brain-derived neurotrophic factor (BDNF; Khaspekov et al., 2004), a neurotrophin reduced in the AD brain (Lee et al., 2005; Peng et al., 2009), which is known to confer protection against excitotoxicity and to promote neurogenesis (Scharfman et al., 2005) and neuronal plasticity; all of these processes play a role in AD. However, there is still no evidence about the implication that such cannabinoid-induced BDNF promotion could have on the cognitive or pathological aspects of AD. Similarly, little is known about the participation of cannabinoid signaling in the impaired function of degradation pathways such as autophagy and ubiquitin–proteasome, which are known to play a relevant role in AD progression. The impairment in these catabolic processes results in accumulation of aggregate-prone proteins, altered mitochondria and other cellular organelles that might exacerbate neurodegenerative process (Nixon et al., 2005; Harris and Rubinsztein, 2011). To date, only one study has reported beneficial effects of cannabinoid-induced autophagy in a model of tauopathy (Casarejos et al., 2013), but this has opened the possibility of exploring in greater detail the involvement of ECS in promoting degradation of toxic components in neurodegenerative diseases. Finally, the effect of cannabinoids on the regulation of cerebral blood flow may contribute to their potential benefits on AD. A number of studies have demonstrated that certain cannabinoids produce vasodilatation of brain blood vessels and increase cerebral blood flow (Ellis et al., 1995; Wagner et al., 2001; Pacher et al., 2005; Iring et al., 2013). Considering that cerebral blood flow in AD contributes to the reduction of oxygen and nutrients in brain (Iadecola, 2004), it can be suggested that treatments improving cerebral perfusion such as cannabinoids are advantageous in AD. Taken together, available information suggest that cannabinoids may have multiple effects on AD by acting not only as anti-oxidant and anti-inflammatory agents, but also modulating a plethora of factors which contribute to the pathogenesis of AD as altered Aβ metabolism, autophagy, trophic factor deficiencies, and impaired blood flow.

## CONCLUSIONS AND THERAPEUTIC IMPLICATIONS

**FIGURE 1:**
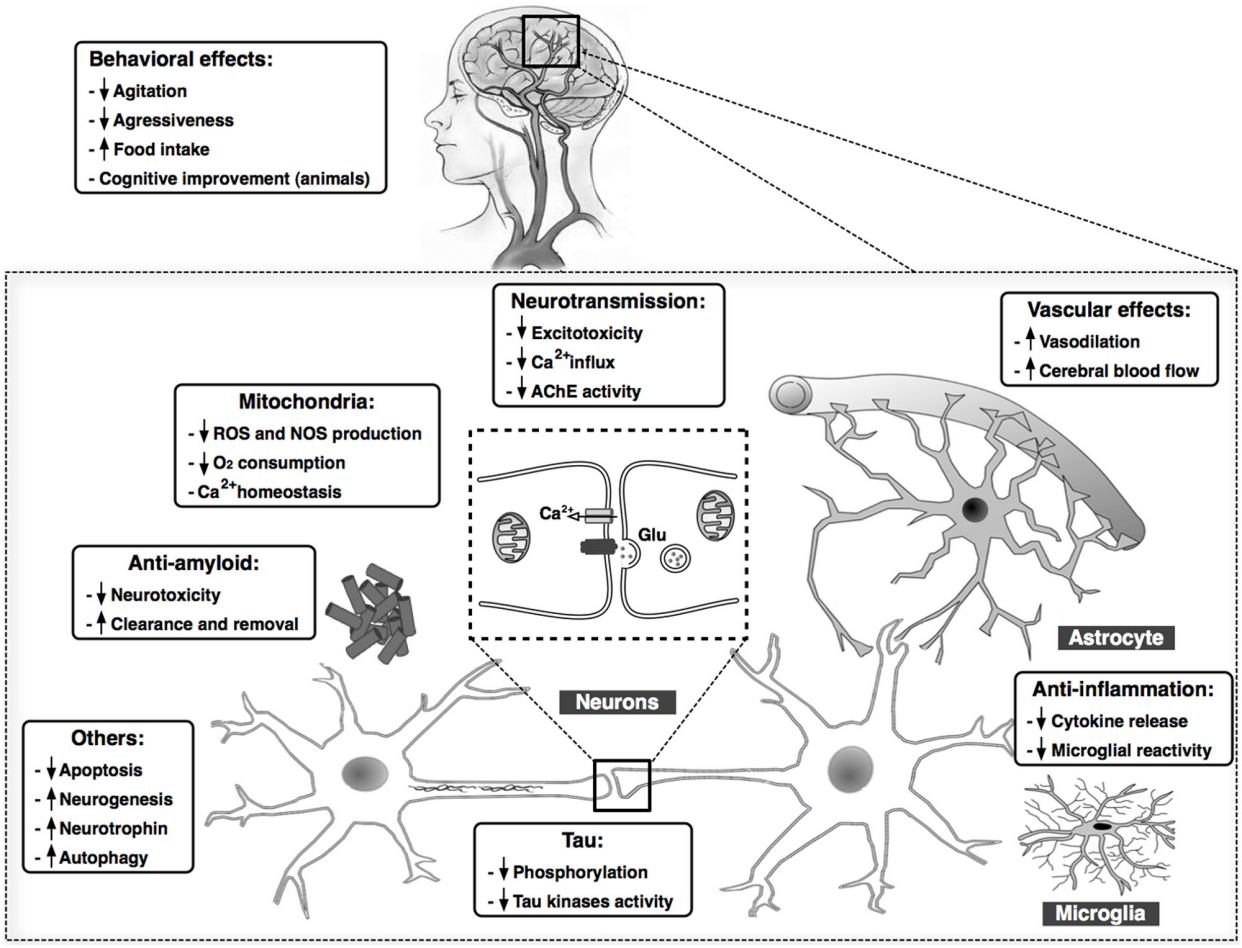
**Summary of the main findings demonstrating beneficial effects of cannabinoid compounds in AD models.** Cannabinoids may target in parallel several processes that play key roles in AD, including Aβ and tau aberrant processing, chronic inflammatory responses, excitotoxicity, mitochondrial dysfunction, and oxidative stress, among others. Clinical data also reveal an improvement in behavioral in patients with AD after treatment with cannabinoids.

Considering the numerous complex pathological mechanisms involved in the progression of AD, treatments targeting a single causal or modifying factor offer limited benefit. Cannabinoids, however, exhibit pleiotropic activity, targeting in parallel several processes that play key roles in AD, including Aβ and tau aberrant processing, neuroinflammation, excitotoxicity, mitochondrial dysfunction, and oxidative stress. Cannabinoids improve behavioral disturbances, as well. These effects are summarized in **Figure 1**. Then, because of these widespread properties of cannabinoid compounds, targeting the ECS could represent a unique and reliable opportunity to advance toward an effective therapy against the AD. Moreover, cannabinoids might represent a safe low-cost therapy, with their natural origin and low side effects profile. From our point of view, the success of cannabinoid-based therapy in AD could be increased taking into account two important aspects: (i) the use of a combination of compounds that cover the whole spectrum of therapeutic properties described for cannabinoids, i.e., combination of CB_1_ and CB_2_ receptors agonists plus CBD, which presents interesting neuroprotective properties spite of its mechanism of action remaining poorly understood, and (ii) the early initiation of the treatment in the neurodegenerative process, which ensures the integrity of the ECS target components and increases the possibility of curbing the exponential degenerative progression toward dementia.

The main concerns regarding the use of cannabis derivatives in medicine are related with the psychoactivity of some cannabinoids, especially Δ^9^-THC, which may disrupt short-term memory, working memory, and attention skills mainly acting through CB_1_ receptors, as well as with the potential Δ^9^-THC dependence occurring after long-term use. However, the therapeutic effects of cannabinoids must be clearly dissociated from the risks of abuse and addiction linked to the recreational use of cannabis derivatives. First, the CB_1_ agonists with potential psychoactivity used in experimental models to demonstrate the therapeutic properties were administered at doses substantially lower than those producing psychoactive effects and cannabis dependence (Maldonado et al., 2011). Second, the preferred therapeutic cannabinoid combination includes CBD, which is known to mitigate the negative consequences on cognition of Δ^9^-THC administration (Fadda et al., 2004), and therefore insure the avoidance of such undesirable effects. Finally, the brain context in healthy subjects consuming cannabis enriched in Δ^9^-THC for recreational purposes is completely different from that of AD patients subjected to very determined combinations of cannabinoid species, in terms of ECS organization and neuronal signaling. In conclusion, in light of the polyvalent properties for the treatment of AD and the limited side effects exhibited by these compounds, progress toward a clinical trial to test the capacity of cannabinoids to curb this neurodegenerative disease seems to be fully justified.

## Conflict of Interest Statement

The authors declare that the research was conducted in the absence of any commercial or financial relationships that could be construed as a potential conflict of interest.
